# A Case of Immunoglobulin A (IgA)-Dominant Infection-Related Glomerulonephritis Treated With Plasmapheresis

**DOI:** 10.7759/cureus.22916

**Published:** 2022-03-07

**Authors:** Said Al Zein, Ali Shueib, Muhannad Alqudsi

**Affiliations:** 1 Department of Medicine, University of Pittsburgh, Coudersport, USA; 2 Department of Nephrology, Ochsner Health System, New Orleans, USA; 3 Department of Nephrology, The University of Queensland, Saint Lucia, AUS

**Keywords:** iga-dominant infection-related glomerulonephritis, plasma exchange, plasmapheresis, staphylococcus-associated glomerulonephritis, postinfectious glomerulonephritis

## Abstract

Immunoglobulin A (IgA)-dominant infection-related glomerulonephritis (IRGN) is mostly associated with Staphylococcal or other bacterial infections like Streptococcus and Gram-negative bacilli. Antibiotics are the cornerstone of treatment in these cases. When the bacterial infection can’t be recognized or IRGN persists despite treating the underlying infection, controlling the kidney injury becomes cumbersome and lacks a strong evidence-based approach. In this report, we describe a 38-year-old male patient with a history of polysubstance abuse and chronic hepatitis B and hepatitis C infections who presented with acute kidney injury and nephrotic syndrome due to IgA-dominant IRGN without an active concurrent bacterial infection who responded well to plasmapheresis.

## Introduction

Immunoglobulin A (IgA)-dominant infection-related glomerulonephritis (IRGN) is typically caused by circulating immune complexes that deposit in the glomeruli with dominance or co-dominance of IgA [1]. Prolonged infections, most commonly due to Staphylococcus, have higher chances of forming immune complexes resulting in renal inflammation [1]. Most of the IgA-dominant IRGN cases occur in adults and are associated with an infection at the time of renal injury. Therefore, antibiotics have been the cornerstone of treatment [1,2]. There is no specific treatment for IgA-dominant IRGN without identifiable infection. Strong data regarding the use of immune suppression in these cases is lacking, although the use of steroids with or without cyclophosphamide has been reported with inconsistent outcomes [2].

## Case presentation

A 38-year-old male patient presented to the emergency room with worsening anasarca and dyspnea for six weeks. His past medical history was significant for polysubstance abuse, cirrhosis, chronic hepatitis C virus (HCV) infection, and chronic hepatitis B virus (HBV) infection and was on tenofovir; he was diagnosed with all four years prior to presentation. The patient denied any other symptoms, no hematuria, no recent infections or rashes prior to presentation. He also denied any recent nonsteroidal anti-inflammatory drugs, antibiotics, or illicit drug use.

In the ER, his blood pressure was 139/79 mmHg, heart rate was 80 beats per minute, respiratory rate was 16 breaths per minute, temperature was 98.8°F (37.1°C), and oxygen saturation was 98%. On physical examination, his chest was clear to auscultation without any crackles. He had abdominal distention, significant lower extremities, and scrotal edema.

His initial lab tests showed creatinine of 2.7 mg/dL which was a significant increase from a baseline of 1 mg/dL one month prior to presentation. His urine test showed proteinuria in addition to acanthocytes and red blood cell casts. The relevant initial lab tests are summarized in Table 1. His kidney ultrasound was unremarkable. The patient was admitted to the hospital and was started on diuretics. 

**Table 1:** Summary of initial lab tests of the patient. HPF: high power field

Test	Result	Reference range
Blood urea nitrogen (BUN)	49 mg/dL	6-20 mg/dL
Creatinine	2.7 mg/dL	0.5-1.2 mg/dL
Aspartate aminotransferase (AST)	64 U/L	10-42 U/L
Alanine aminotransferase (ALT)	35 U/L	10-60 U/L
White blood cell count	7.5 k/uL	4.8-10.8 k/uL
Hemoglobin	12.8 g/dL	14-18 g/dL
Complement C3	37 mg/dL	55-120 mg/dL
Complement C4	12 mg/dL	10-40 mg/dL
Cryoglobulins	Absent	Absent
Anti-nuclear antibodies (ANA)	Negative	Negative
Anti-double-stranded DNA (anti-dsDNA)	Negative	Negative
Urine protein to creatinine ratio	Too high to calculate	22-128 mg/g
Urine red blood cells (RBC)	> 100/HPF	≤2 /HPF
Urine white blood cells (WBC)	0 /HPF	≤5 /HPF
Urine microscopy	Acanthocytes, red blood cell casts and white blood cell casts	

**Figure 1:**
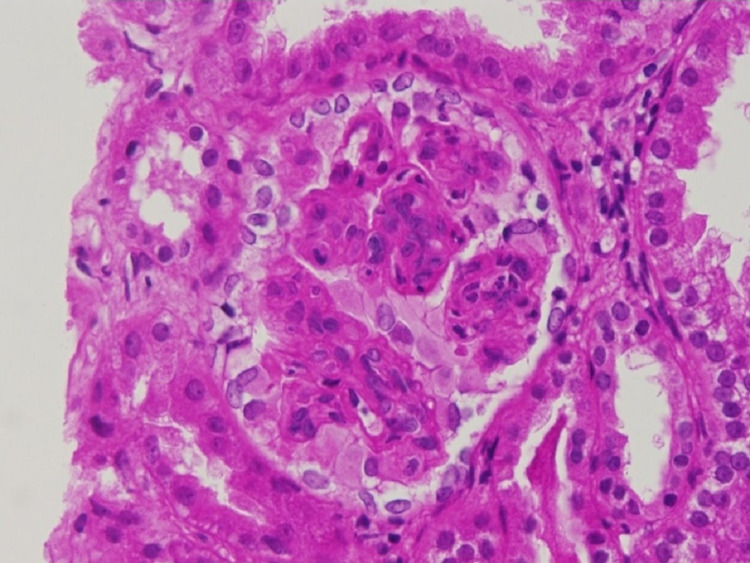
Endocapillary hypercellularity (light microscopy) showing proliferative glomerulonephritis on hematoxylin and eosin (H&E) stain. Numerous neutrophils within glomerular capillaries.

**Figure 2:**
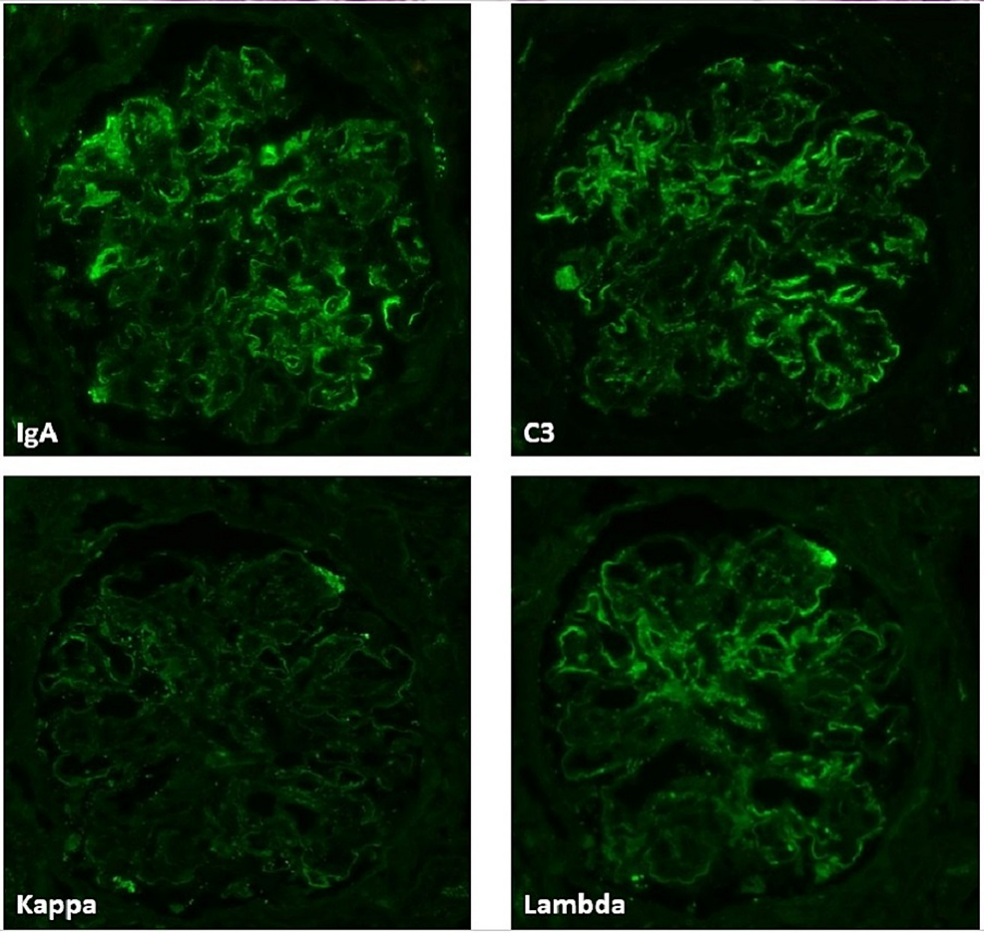
Immunofluorescence microscopy (IF) showing bright mesangial and capillary loop staining for IgA (+1-2) and C3 (+2); stronger lambda (+1) than kappa (trace) staining.

**Figure 3:**
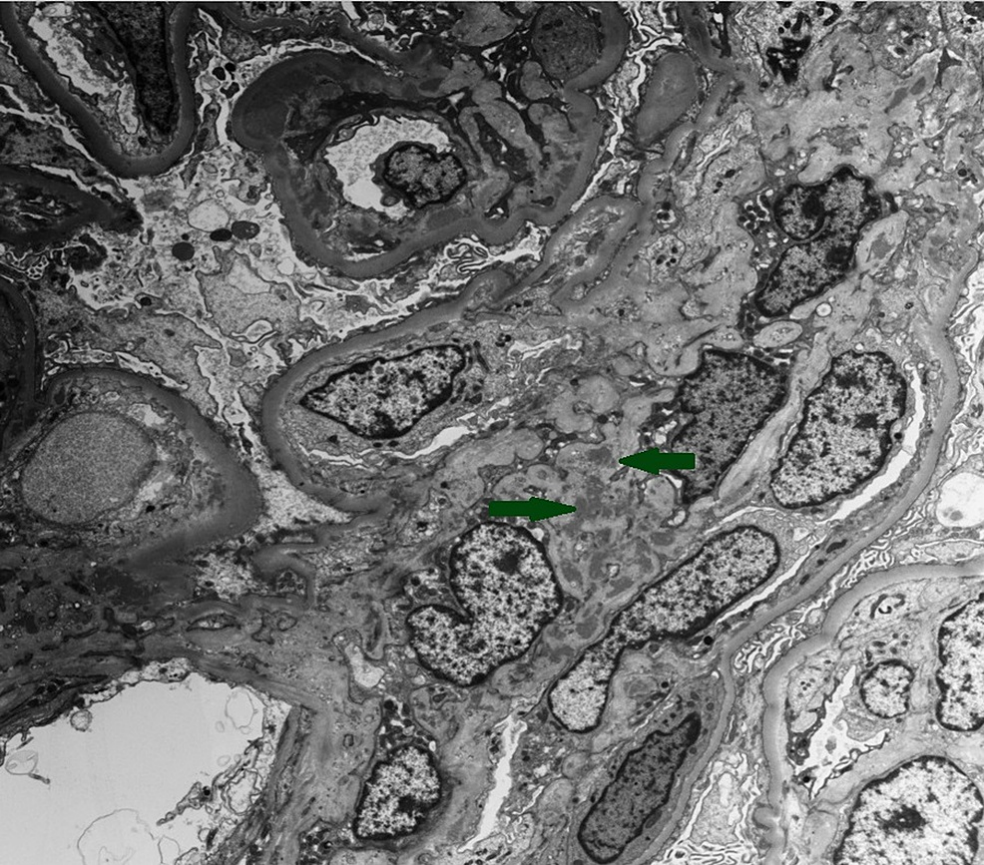
Electron microscopy showing frequent mesangial immune deposits (arrows).

**Figure 4:**
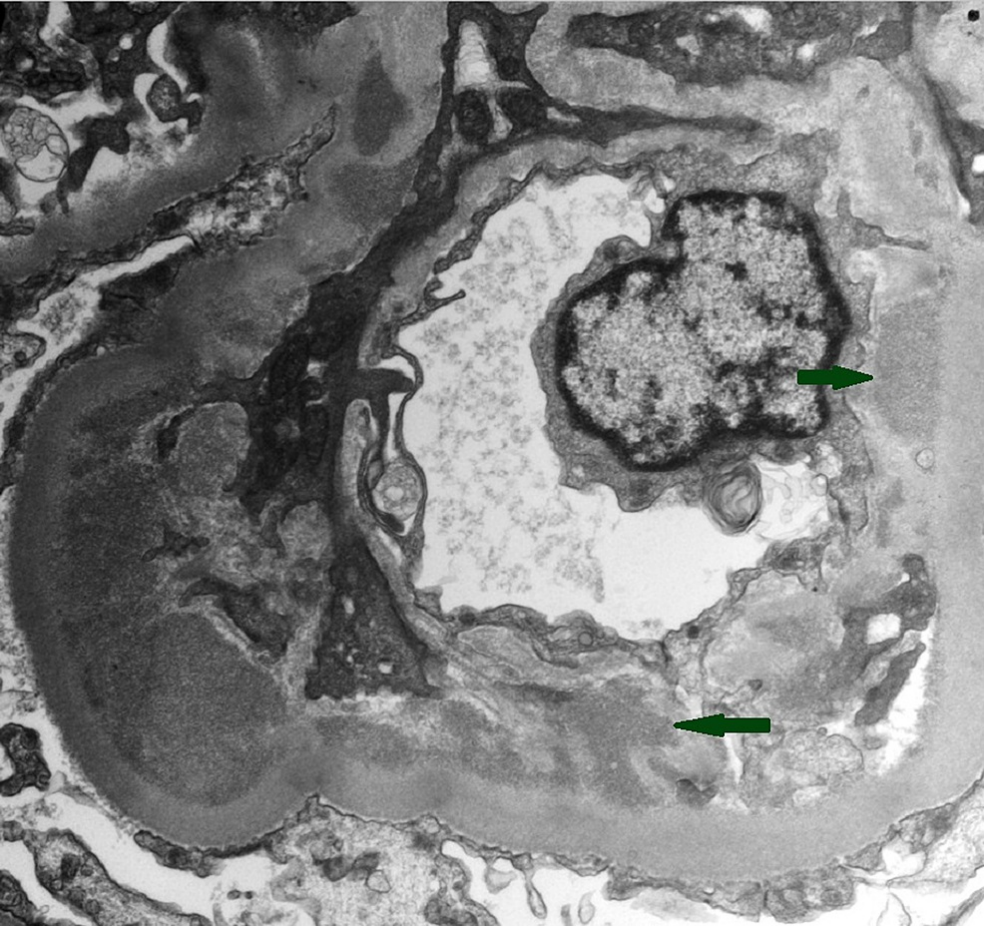
Electron microscopy showing frequent small to medium subendothelial immune deposits (arrows).

A kidney biopsy was performed which revealed 19 glomeruli, six were globally sclerosed and the remaining had endocapillary hypercellularity without crescents (Figure 1). On immune-florescence, IgA was the dominant immunoglobulin but C3 had stronger staining (Figure 2). Electron microscopy revealed frequent mesangial (Figure 3) and subendothelial immune deposits (Figure 4). 

Extensive workup to identify an infection was performed, which came out negative including blood, sputum, urine, and ascites cultures. HCV and HBV viral loads were undetectable and human immunodeficiency virus (HIV) serologies were negative. Trans-thoracic echocardiogram did not show endocarditis. Trans-esophageal echocardiogram was not indicated according to Duke criteria and therefore was not done.

His creatinine continued to rise during the hospital stay. Plasmapheresis was initiated when creatinine reached 4.7 mg/dL. A total of six sessions were performed. His creatinine started improving on the second day of plasmapheresis and reached 2.4 mg/dL 10 days later. Additionally, his albumin improved to 4.1 g/dL and urine protein to creatinine ratio dropped to 3.5 g/g. The major events and procedures during his five weeks hospital stay are explained chronologically in Table 2. The patient's creatinine continued to improve after discharge and reached 1.5 mg/dL four months later.

**Table 2:** Major events and procedures of the patient during five weeks of hospital stay are explained chronologically. Creatinine levels on certain dates are reported as ranges to avoid an unnecessarily long list of data.

Date	Creatinine (mg/dL)	Events
April 22	1	-
May 16	2.7	Admission to the hospital
May 17 until May 24	2.7-3.2	-
May 25	3.5	Biopsy performed
May 26	3.2	-
May 27 until June 8	3.6-4.3	-
Jun 9	4.6	-
June 10	4.7	1st plasmapheresis
June 11	4.4	2nd plasmapheresis
June 12	4.1	3rd plasmapheresis
June 13	3.7	4th plasmapheresis
June 14	3.3	-
June 15	2.9	5th plasmapheresis
June 16	2.8	-
June 17	2.8	6th plasmapheresis
June 18	2.7	-
June 19	2.5	-
June 20	2.4	-
June 21	2.4	Discharge from the hospital

## Discussion

Bacterial infection-related glomerulonephritis has been classically considered an immune complex glomerular disease that occurs in children few weeks after the resolution of a group A beta-hemolytic streptococcus infection, and for this reason, it has been referred to as post-infectious glomerulonephritis or post-streptococcal glomerulonephritis (PSGN). Most children are asymptomatic. Other presentations range from microscopic hematuria to a full nephritic picture and only in rare cases rapidly progressive glomerulonephritis [3,4].

The rates of PSGN have been declining over the past few decades with the use of antibiotics and improved management of infections. On the other hand, the rates of IRGN have been increasing in the adult population. A bacterial infection is often identified at the time of presentation of IRGN in adults. Hence, the terms infection-related and infection-associated glomerulonephritis are usually used in this population [2]. The presentation is more severe in adults, and it is likely to occur in the elderly population and in patients with chronic illnesses such as diabetes, IV drug abuse, or malignancy [2,5]. 

The pathological pattern of IRGN is typically an acute proliferative and exudatives glomerulonephritis. Light microscopy typically shows diffuse endocapillary proliferative glomerulonephritis with infiltrating neutrophils and more than 50% of glomeruli showing occlusion of the peripheral capillaries by endocapillary hypercellularity. Glomerular immune deposits are seen on immunofluorescence in a granular, predominantly capillary wall distribution pattern and stain for either C3 alone or C3 with IgG or IgA. Subepithelial “hump-shaped” and subendothelial deposits can be seen on electron microscopy (EM) [5].

IgA dominance or co-dominance is common in adults with staphylococcal infections. IgA-dominant IRGN was first described in 2003 in a series of adult patients with diabetic nephropathies [6]. Other pathological patterns of IRGN are crescentic and necrotizing glomerulonephritis which is usually seen in infective endocarditis [7,8] and membranoproliferative glomerulonephritis (MPGN) pattern in shunt nephritis [9,10].

Our patient had endocapillary hypercellularity along with a dominant IgA and C3 mesangial and capillary loop staining. Frequent mesangial and subendothelial immune deposits were seen on electron microscopy. The absence of subepithelial humps on EM does not rule out IRGN as this finding was only observed in 63.5% of patients in a review of 78 patients with IgA-dominant IRGN [11] and as low as 31% of patients in another report [12]. One important differential diagnosis to consider is IgA nephropathy. However, the endocapillary hypercellularity, strong C3 staining, and low C3 serum complement level make the diagnosis of IgA nephropathy less likely [6].

Patients with IgA-dominant IRGN usually have chronic illnesses in addition to the concurrent infection at the time of presentation. In a review of 78 patients with biopsy-proven IgA-dominant IRGN, 70% of patients had Staphylococcus. Other reported organisms included human immunodeficiency virus (HIV), Streptococcus, Klebsiella, *Escherichia coli*, Rickettsia, *Acinetobacter baumannii*, *Chlamydophila pneumoniae*, and hepatitis A virus. No pathogen was detected in 10 patients [11]. 

Clearing the infection has been considered the cornerstone of therapy. This is done by the appropriate use of antibiotics and surgery if indicated [1,2]. Additional needed treatments are salt restriction, anti-hypertensive drugs, and diuretics to control hypertension and volume overload. Angiotensin-converting enzyme (ACE) inhibitors or angiotensin receptor blockers (ARB) are usually used after acute glomerulonephritis has resolved for management of hypertension, especially in patients with persistent proteinuria above 1000 mg per day [4,6].

There are limited data on the use of immune suppression in IRGN. Steroids have been used in several reports without consistent outcomes [13-17]. Cyclophosphamide use along with steroids has been reported in crescentic IRGN without reliable benefit either [18,19]. There are no specific treatment recommendations for cases of IgA-dominant IRGN without an identifiable concurrent bacterial infection.

The use of plasmapheresis in this immune complex-mediated disease along with steroids has been reported in a patient with infective endocarditis-related crescentic glomerulonephritis with a good response [20]. Extensive workup was performed for our patient to rule out any bacterial infection and despite the absence of a bacterial infection, the renal biopsy was suggestive of IgA-dominant IRGN as discussed above. We used plasmapheresis without steroids and the patient’s creatinine improved promptly.

## Conclusions

Infection-related glomerulonephritis is a condition caused by immune complex deposits in the glomeruli. The incidence of this condition in adults is becoming more common and is usually associated with a concurrent bacterial infection in this population. Limiting additional immune complex formation by eradication of the bacterial infection is the main treatment strategy. In patients without identifiable infection, such as our patient, or patients with persistent IRGN despite appropriate management of the infection, plasmapheresis should be considered.

## References

[1] Nadasdy T, Hebert LA (2011). Infection-related glomerulonephritis: understanding mechanisms. Semin Nephrol.

[2] Nasr SH, Radhakrishnan J, D'Agati VD (2013). Bacterial infection-related glomerulonephritis in adults. Kidney Int.

[3] Sanjad S, Tolaymat A, Whitworth J, Levin S (1977). Acute glomerulonephritis in children: a review of 153 cases. South Med J.

[4] Rodríguez-Iturbe B (1984). Epidemic poststreptococcal glomerulonephritis. Kidney Int.

[5] Nasr SH, Fidler ME, Valeri AM (2011). Postinfectious glomerulonephritis in the elderly. J Am Soc Nephrol.

[6] Nasr SH, Markowitz GS, Whelan JD (2003). IgA-dominant acute poststaphylococcal glomerulonephritis complicating diabetic nephropathy. Hum Pathol.

[7] Majumdar A, Chowdhary S, Ferreira MA, Hammond LA, Howie AJ, Lipkin GW, Littler WA (2000). Renal pathological findings in infective endocarditis. Nephrol Dial Transplant.

[8] Boils CL, Nasr SH, Walker PD, Couser WG, Larsen CP (2015). Update on endocarditis-associated glomerulonephritis. Kidney Int.

[9] Vella J, Carmody M, Campbell E, Browne O, Doyle G, Donohoe J (1995). Glomerulonephritis after ventriculo-atrial shunt. QJM.

[10] Kiryluk K, Preddie D, D'Agati VD, Isom R (2008). A young man with Propionibacterium acnes-induced shunt nephritis. Kidney Int.

[11] Bu R, Li Q, Duan ZY, Wu J, Chen P, Chen XM, Cai GY (2015). Clinicopathologic features of IgA-dominant infection-associated glomerulonephritis: a pooled analysis of 78 cases. Am J Nephrol.

[12] Satoskar AA, Suleiman S, Ayoub I (2016). Staphylococcus infection-associated GN - spectrum of IgA staining and prevalence of ANCA in a single-center cohort. Clin J Am Soc Nephrol.

[13] Kikuchi H, Aoyagi M, Nagahama K (2014). IgA-dominant postinfectious glomerulonephritis associated with Escherichia coli infection caused by cholangitis. Intern Med.

[14] Nagaba Y, Hiki Y, Aoyama T (2002). Effective antibiotic treatment of methicillin-resistant Staphylococcus aureus-associated glomerulonephritis. Nephron.

[15] Satoskar AA, Nadasdy G, Plaza JA, Sedmak D, Shidham G, Hebert L, Nadasdy T (2006). Staphylococcus infection-associated glomerulonephritis mimicking IgA nephropathy. Clin J Am Soc Nephrol.

[16] Handa T, Ono T, Watanabe H, Takeda T, Muso E, Kita T (2003). Glomerulonephritis induced by methicillin-sensitive Staphylococcus aureus infection. Clin Exp Nephrol.

[17] Pérez A, Torregrosa I, D'Marco L (2021). IgA-dominant infection-associated glomerulonephritis following SARS-CoV-2 infection. Viruses.

[18] Raff A, Hebert T, Pullman J, Coco M (2005). Crescentic post-streptococcal glomerulonephritis with nephrotic syndrome in the adult: is aggressive therapy warranted?. Clin Nephrol.

[19] Zent R, Smit RV, Duffield M, Cassidy MJ (1994). Crescentic nephritis at Groote Schuur Hospital, South Africa--not a benign disease. Clin Nephrol.

[20] Malhotra K, Yerram P (2019). Plasmapheresis and corticosteroids in infective endocarditis-related crescentic glomerulonephritis. BMJ Case Rep.

